# Factors predicting improved compliance towards colonoscopy in individuals with positive faecal immunochemical test (FIT)

**DOI:** 10.1002/cam4.4275

**Published:** 2021-09-14

**Authors:** Tian‐Zhi Lim, Jerrald Lau, Gretel Jianlin Wong, Lavine Yen‐Ting Tan, Yu‐Jing Chang, Karthiga Natarajan, Huso Yi, Mee‐Lian Wong, Ker‐Kan Tan

**Affiliations:** ^1^ Division of Colorectal Surgery University Surgical Cluster National University Health System Singapore; ^2^ Department of Surgery Yong Loo Lin School of Medicine National University of Singapore Singapore; ^3^ Saw Swee Hock School of Public Health National University of Singapore Singapore

**Keywords:** colorectal cancer, compliance, evaluation, health belief model, screening

## Abstract

**Objectives:**

Follow‐up colonoscopy after a positive faecal immunochemical test (FIT) in any colorectal cancer (CRC) screening programme is integral. However, many individuals who had a positive FIT declined colonoscopy subsequently. This study aims to uncover the predictors on completion of colonoscopy using the Health Belief Model (HBM) between individuals who complete and those who did not after a positive FIT.

**Methods:**

A mixed‐method study comprising qualitative semi‐structured interviews followed by a locally validated questionnaire in Singapore was prospectively administered via telephone interview to average risk individuals with positive FIT results from a cohort of the national FIT screening database referred for follow‐up colonoscopic evaluation.

**Results:**

A total of 394 individuals, with a median age of 66 years (range, 46–89 years), were recruited. Fifty percent completed follow‐up colonoscopic evaluation and formed the “doers” group. All participants demonstrated high knowledge of symptoms of CRC and awareness and qualitative responses were aligned to the various HBM domains. Using multi‐variable analysis, doers felt that medical recommendations (odds ratio [OR], 2.39, 95% confidence interval [CI]: 1.23–4.63, *p* = 0.01) and mainstream media publicity (OR, 2.16, 95% CI: 1.09–4.26, *p* = 0.026) were important. Non‐doers showed positive association with perceived barriers such as cost (OR, 2.15, 95% CI: 1.10–4.20, *p* = 0.026) and inconvenience (OR, 3.44, 95% CI: 1.50–7.89, *p* = 0.004).

**Conclusions:**

Identified factors such as tackling perceived barriers, public health education and active promotion by medical physicians, family and friends could help guide subsequent interventions to improve compliance of individuals with positive FIT to undergo follow‐up colonoscopy.

## Abstract 2

Only a third of FIT‐positive individuals go for follow‐up colonoscopy in Singapore. This is an Asian study comparing knowledge and attitudes of compliant and non‐compliant FIT‐positive individuals towards follow‐up colonoscopy.

## 1 INTRODUCTION

Colorectal cancer (CRC) is the top cancer in Singapore. From 2013 to 2017, a total of 10,634 new cases of CRC were diagnosed.^1^ CRC is one of the few cancers where cancer screening has been shown to be associated with superior oncological outcomes.^2^ In Singapore, the national CRC screening programme advocates the faecal immunochemical test (FIT) as one of the screening modalities for average risk residents aged 50 years and above.^3^ Should the FIT be positive, these individuals will be referred for consideration of colonoscopy at their nearest public hospital.

Unfortunately, not all patients with a positive FIT will undergo a colonoscopy. The 2010 Singapore National Health Survey^4^ reported that only a third of those with positive FIT results actually underwent colonoscopy. Some of the postulated reasons for poor uptake of colonoscopy included inconvenience, accessibility, cost, denial and poor overall awareness of CRC.^5^, ^6^

While much effort has been made to better understand the factors that influence CRC screening behaviour, less has been performed to evaluate the knowledge, barriers and facilitators contributing to completion of colonoscopy after a positive FIT result, which ironically is a much higher risk group than the average population. Not surprisingly, most of the work looking on this issue of non‐compliance has focused on the individuals who were not compliant to the recommendation of colonoscopy.^7^ Few studies, if any, have compared the differences between individuals who completed (doers) and those who did not (non‐doers).

In light of the above considerations, this study aimed to uncover the knowledge, barriers and facilitators influencing the decision to complete the recommended follow‐up colonoscopy in a nationally representative cohort of FIT‐positive individuals in Singapore.

## 2 METHODOLOGY

### 2.1 Theoretical framework

The Health Belief Model (HBM) has been commonly applied to explain intra‐personal decision‐making processes on a wide range of health behaviours, including vaccination and screening.^8^, ^9^, ^10^ *The HBM consists of five domains: perceived benefits, perceived barriers, perceived susceptibility, perceived severity and the presence of cues to action*.^11^, ^12^ These domains influence health behaviour and/or intention, often alongside other intrapersonal modifying factors (e.g. age, gender and health literacy).

### 2.2 Study population and sample

The study population was derived from a cohort comprising all Singapore residents who had (i) undergone a FIT investigation via the national CRC screening programme administered by the Singapore Cancer Society (SCS) in the (ii) years 2017–2019 inclusive, (iii) had received at least one positive FIT result and (iv) were referred to a public hospital for follow‐up consultation and colonoscopy with a gastrointestinal specialist. SCS is a local non‐profit charity organisation recognised by the Ministry of Health (MOH) to fight for a cancer‐free community with the mission to minimise cancer and maximise lives through cancer programmes (i.e. National CRC screening programme).

The list of patients was compiled from the national CRC screening registry provided by the SCS. We defined individuals as “doers” if they had completed the recommended follow‐up colonoscopy at the point of recruitment, and as “non‐doers” if they had not. The cohort was then divided into two lists based on doer or non‐doer status, and simple random sampling was used to prospectively recruit the sample from the study population.

Sample size was calculated using the rule of 10 outcome events per predictor variable.^13^, ^14^ With the intention to have up to 20 variables in the eventual multi‐variable model, approximately 200 participants were needed to provide sufficient power for each group (i.e. doers and non‐doers).

### 2.3 Study measures

A mixed‐method study design was adopted using a semi‐structured interview guide and a locally validated HBM questionnaire. Aside from collecting demographics data and answers to generic questions pertinent to the knowledge of CRC and CRC screening, we also administered a locally validated questionnaire based on the HBM.^15^ The semi‐structured interview was carried out in accordance to the interview guide by trained interviewers while the questionnaire comprised three sections (i.e. socio‐demographics, knowledge of CRC and CRC screening and 28 item in 5 HBM constructs––perceived severity, perceived susceptibility, perceived benefits, perceived barriers and cues to actions).

### 2.4 Study procedure

The survey was administered over telephone by three trained interviewers (LYT, YC and KN) after obtaining informed consent. A non‐response was defined as three separate unsuccessful attempts to contact the same individual on three different days at various time points, or a decline to participate response from the individual, whichever was reached first.

### 2.5 Statistical analyses

Qualitative data were transcribed and thematically analysed using Braun and Clarke's interpretative strategies.^16^ All HBM scale‐based items were assessed for internal consistency using Cronbach’s alpha. All analyses were then stratified comparatively into doers versus non‐doers. Univariate analyses utilised Pearson chi‐squared tests to compare between categorical variables and Mann–Whitney *U* tests to compare non‐parametric continuous variables between groups. Multi‐variable logistic regression analysis was conducted to examine associations between the HBM subscale items and follow‐up colonoscopy compliance status, controlling for participant sociodemographics. Although univariate analyses revealed that there were no significant differences between doer and non‐doer groups, we included all baseline sociodemographic factors into the multi‐variable analysis as classical confounders. The results of this analysis were represented by adjusted odds ratios (aORs) and presented with 95% confidence intervals (CIs), using a two‐tailed *p*‐value of <0.05 to denote statistical significance. All statistical analyses were performed using IBM SPSS Statistics for Windows, version 20 (IBM Corp.).

### 2.6 Research ethics

Ethical approval to conduct this study was obtained. Verbal informed consent was sought and acquired from all participants before data collection.

## 3 RESULTS

### 3.1 Sample sociodemographics

A total of 394 out of 824 (response rate of 47.8%) eligible individuals (i.e. FIT‐positive individuals who defaulted on their follow‐up) agreed to participate in the study. Two hundred (50.8%) of them completed the colonoscopic evaluation and formed the doer group. The median age of the participants was 66 years (range = 46–89 years) with a slight majority of male participants (53.8%). Sociodemographic characteristics were comparable between the doer and non‐doer groups, as seen in Table 1. The interviews lasted about 30–40 min.

**TABLE 1:** Sociodemographic characteristics between doer and non‐Doer

Characteristics	Doer (%) (*N* = 200)	Non‐doer (%) (*N* = 194)	Analysis (*p*‐value, odds ratio)
Median age, years (range)	66 (51–89)	67 (46–89)	0.503 (*p*‐value)
Age distribution			0.274 (*p*‐value)
<60	45 (22.5)	48 (24.7)	
60–70	90 (45)	72 (37.1)	
>70	65 (32.5)	74 (38.1)	
Gender			0.543 1.15 (95% CI 0.77–1.71)
Male	111 (55.8)	101 (52.3)	
Female	88 (44.2)	92 (47.7)	
Race			0.840 (*p*‐value)
Chinese	183 (92)	179 (93.2)	
Malay	6 (3)	5 (2.6)	
Indian	6 (3)	5 (2.6)	
Others	4 (2)	3 (1.6)	
Religion			0.975 (*p*‐value)
No religion	51 (25.5)	53 (27.7)	
Buddhism	69 (34.5)	64 (33.5)	
Christianity	62 (31)	60 (31.4)	
Islam	8 (4)	5 (2.6)	
Hinduism	3 (1.5)	2 (1)	
Taoism	4 (2)	3 (1.6)	
Others	3 (1.5)	4 (2.1)	
Occupation			0.208 (*p*‐value)
Working	77 (38.5)	68 (35.2)	
Unemployed	24 (12)	20 (10.4)	
Retired	90 (45)	102 (52.8)	
Others	9 (4.5)	3 (1.6)	
Income			0.762 (*p*‐value)
$0	1 (0.5)	4 (2.2)	
<$2000	138 (71.5)	128 (69.2)	
$2000–$4000	28 (14.5)	26 (14.1)	
$4000–$6000	12 (6.2)	14 (7.6)	
$6000–$8000	8 (4.1)	6 (3.2)	
>$8000	6 (3.1)	7 (3.8)	
Years of schooling			0.464 (*p*‐value)
None	3 (1.5)	4 (2.1)	
<7	30 (15.1)	19 (10)	
7–13	74 (37.2)	78 (41.1)	
>13 (Tertiary)	92 (46.2)	89 (46.8)	
Housing			0.079 (*p*‐value)
HDB (1–3 rooms)	29 (14.8)	31 (16.9)	
HDB (4–5 rooms)	95 (48.5)	77 (42.1)	
Executive flats	17 (8.7)	6 (3.3)	
Condominium	28 (14.3)	35 (19.1)	
Landed property	27 (13.8)	34 (18.6)	
Marital status			0.204 (*p*‐value)
Single	9 (4.6)	16 (8.4)	
Married	168 (85.3)	163 (85.3)	
Divorced	13 (6.6)	6 (3.1)	
Widowed	7 (3.6)	6 (3.1)	

#### 3.1.1 Qualitative component

Three main themes emerged from the analysis. Excerpts of the transcript can be found in Data S1.

##### *(1)* High level of CRC awareness amongst average risk individuals.

**FIGURE 1:**
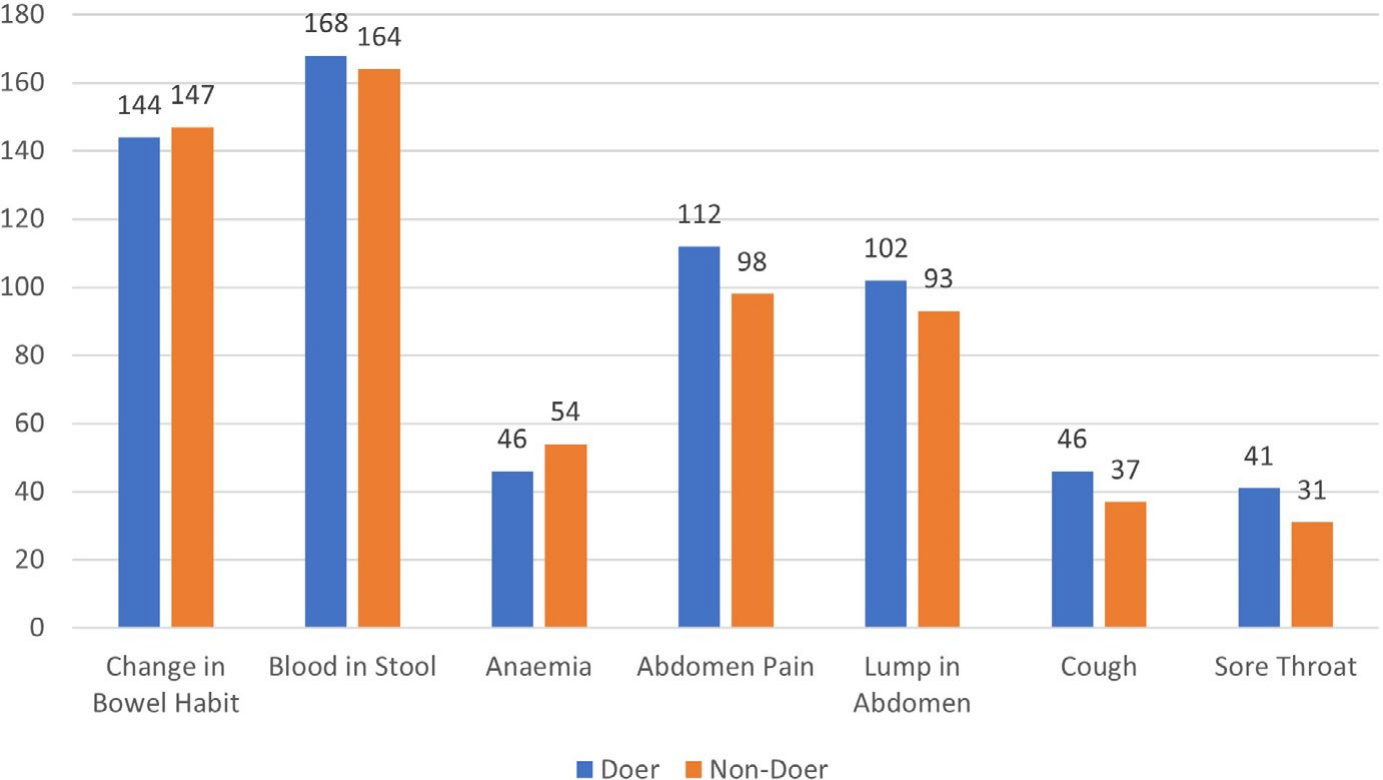
Source of colorectal cancer screening awareness

The responses were generally aligned to the various HBM domains showcasing high level of CRC awareness. When asked about the need for repeated calling and reminders, doers were more likely to express that repeated calls and reminders were not necessary since they already had the information. Non‐doers, however, are more likely to prefer these reminders as they could have been busy or plain lazy. Respondents were generally aware of the main sources of information regarding CRC screening. Responses were similar to sources featured in Figure 1

##### *(2)* Health seeking behaviour determined by degree of personal motivation.

Most respondents commented that it was the value of their lives which prompted them to seek medical attention and to check that they are in pink of health. In the non‐doer group, it is not the fear of the colonoscopy procedure, nor the worry of knowing the diagnosis, or the assumed embarrassment of the procedure that could account for their non‐compliance. Structural barriers such as inconvenience of the current processes and concerns over the colonoscopy procedure itself was less of a concern. However, worries over bowel preparation and cost implications of the procedure featured strongly in the participants’ replies.

##### *(3)* Importance of social support and medical professional advocacy.

The importance of family and close friends for social support and high regard of healthcare professional medical recommendation were deemed beneficial and cannot be understated when reaching out to the public. They form strong support network to reinforce the need for CRC screening.

#### 3.1.2 Quantitative component

##### Knowledge on CRC

**FIGURE 2:**
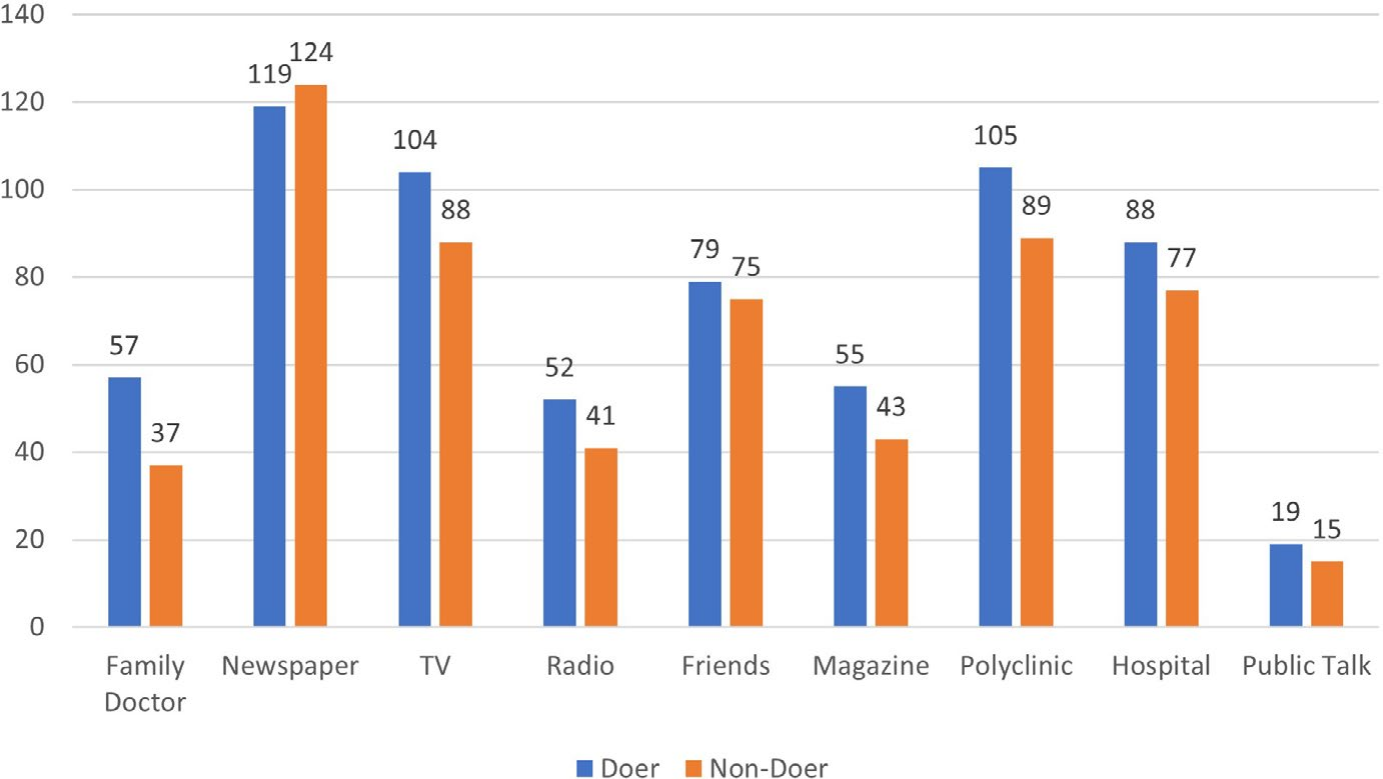
Knowledge on presenting symptoms of colorectal cancer

The majority of participants (80.2%) were aware that CRC was amongst the top three cancers in Singapore. Most participants knew that change in bowel habits (73.9%) and presence of blood in stools (84.3%) were possible symptoms of CRC (see Figure 2). Participants were also aware of common sources of CRC screening awareness such as (i) medical recommendation (73.9%), (ii) mainstream media (e.g. newspaper and television) (55.2%) and (iii) peer support (39.1%; see Figure 1, Table 2).

**TABLE 2:** Knowledge of colorectal cancer

Characteristics	Doer (%) (*N* = 200)	Non‐doer (%) (*N* = 194)	Analysis (*p*‐value, odds ratio)
Colorectal cancer is amongst the top three cancers in Singapore			
Yes	160 (80.4)	156 (80.4)	1.0
No/do not know	39 (19.6)	38 (19.6)	1.0 (95% CI 0.608–1.647)
Knowledge of symptoms of colorectal cancer:			
A. Change in bowel habit			
Yes	144 (72.4)	147 (75.8)	0.49
No/do not know	55 (27.6)	47 (24.2)	0.84 (95% CI 0.532–1.32)
B. Blood in stools			
Yes	168 (84.4)	164 (85)	0.889
No/do not know	31 (15.6)	29 (15)	0.958 (95% CI 0.55–1.66)
C. Anaemia (low red blood cell count)			
Yes	46 (23.1)	54 (27.8)	0.299
No/do not know	153 (76.9)	140 (72.2)	0.779 (95% CI 0.495–1.23)
D. Abdominal pain			
Yes	112 (56.3)	98 (50.5)	0.267
No/do not know	87 (43.7)	96 (49.5)	1.261 (95% CI 1.09–1.88)
E. Lump in the abdomen			
Yes	102 (51.3)	93 (47.9)	0.545
No/do not know	97 (48.7)	101 (52.1)	1.14 (95% CI 0.77–1.70)
F. Cough			
Yes/do not know	46 (23.1)	37 (19.1)	0.387
No	153 (76.9)	157 (80.9)	1.28 (95% CI 0.78–2.07)
G. Sore throat			
Yes/do not know	41 (20.7)	31 (16)	0.242
No	157 (79.3)	163 (84)	1.37 (95% CI 0.82–2.30)
Knowledge of colorectal cancer prevention:			
A. Do not overeat			
Yes	88 (44.2)	108 (55.7)	0.027
No/do not know	111 (55.8)	86 (44.3)	0.63 (95% CI 0.42–0.92)
B. More vegetables and fruits in diet			
Yes	172 (86.4)	165 (85.5)	0.885
No/do not know	27 (13.6)	28 (14.5)	1.08 (95% CI 0.61–1.91)
C. Do exercise			
Yes	165 (82.9)	154 (79.4)	0.439
No/do not know	34 (17.1)	40 (20.6)	1.26 (95% CI 0.76–2.09)
D. Not to smoke			
Yes	145 (72.9)	138 (71.5)	0.822
No/do not know	54 (27.1)	55 (28.5)	1.07 (95% CI 0.69–1.66)
Awareness of Colonoscopy			
Yes	191 (96)	181 (93.3)	0.268
No/do not know	8 (4)	13 (6.7)	1.71 (95% CI 0.69–4.24)

##### HBM constructs

Cronbach's alphas for the items within each subscale were computed, which ranged from 0.31 to 0.68. Given the low internal consistency of the subscales, the items were analysed separately (see Tables 3 and 4).

**TABLE 3:** Prevalence of domains in health belief model by doer versus non‐doer

Characteristics	Doer (%) (*N* = 200)	Non‐doer (%) (*N* = 194)	Analysis (*p*‐value, odds ratio)
Perceived severity			
CRC will lead to suffering			
Agree	186 (93.9)	175 (90.2)	0.193
Disagree/unsure	12 (6.1)	19 (9.8)	1.68 (95% CI 0.79–3.57)
CRC will lead to death			
Agree	174 (87.9)	160 (82.5)	0.155
Disagree/unsure	24 (12.1)	34 (17.5)	1.54 (95% CI 0.88–2.71)
CRC will affect my Family			
Agree	182 (92.4)	168 (86.6)	0.07
Disagree/unsure	15 (7.6)	26 (13.4)	1.88 (95% CI 0.96–3.66)
CRC will affect my work			
Agree	186 (94.9)	173 (89.2)	0.04
Disagree/unsure	10 (5.1)	21 (10.8)	2.26 (95% CI 1.09–4.93)
CRC will affect my social life			
Agree	181 (91.9)	165 (85.5)	0.055
Disagree/unsure	16 (8.1)	28 (14.5)	1.92 (95% CI 1–3.68)
CRC is expensive to treat			
Agree	134 (68.4)	147 (75.8)	0.115
Disagree/unsure	62 (31.6)	47 (24.2)	0.691 (95% CI 0.443–1.079)
Perceived susceptibility			
I have some/high changed of developing CRC			
Agree	96 (49)	82 (42.3)	0.188
Disagree/unsure	100 (51)	112 (57.7)	1.31 (95% CI 0.88–1.96)
I never worry about getting CRC			
Agree/unsure	99 (50.3)	92 (47.7)	0.614
Disagree	98 (49.7)	101 (52.3)	1.11 (95% CI 0.88–1.65)
It is fated that I will get CRC			
Agree	36 (18.4)	39 (20.2)	0.7
Disagree/unsure	160 (81.6)	154 (79.8)	0.888 (95% CI 0.537–1.47)
I can prevent myself from getting CRC			
Agree	164 (83.2)	165 (85.1)	0.679
Disagree/unsure	33 (16.8)	29 (14.9)	0.87 (95% CI 0.75–1.50)
Perceived benefits			
CRC screening helps to detect cancer early			
Agree	194 (98.5)	187 (96.9)	0.334
Disagree/unsure	3 (1.5)	6 (3.1)	2.07 (95% CI 0.51–8.40)
Early diagnosis of CRC can increase my chances of survival			
Agree	195 (99)	189 (97.4)	0.282
Disagree/unsure	2 (1)	5 (2.6)	2.58 (95% CI 0.55–13.51)
Early diagnosis of CRC can reduce future medical expenses			
Agree	184 (93.4)	179 (92.3)	0.699
Disagree/unsure	13 (6.6)	15 (7.7)	1.19 (95% CI 0.55–2.56)
Perceived barriers			
I rather not know if I had CRC			
Agree	32 (16.2)	23 (11.9)	0.246
Disagree/unsure	165 (83.8)	170 (88.1)	1.43 (95% CI 0.81–2.55)
I am afraid of finding out if I have CRC			
Agree	61 (31)	48 (24.7)	0.178
Disagree/unsure	136 (69)	146 (75.3)	1.36 (95% CI 0.87–2.13)
CRC screening is expensive			
Agree	102 (52)	106 (54.6)	0.613
Disagree/unsure	94 (48)	88 (45.4)	0.9 (95% CI 0.605–1.34)
Colonoscopy is dangerous			
Agree	26 (13.2)	31 (16)	0.475
Disagree/unsure	171 (86.8)	163 (84)	0.799 (95% CI 0.455–1.404)
Colonoscopy is painful			
Agree	38 (19.3)	43 (22.2)	0.533
Disagree/unsure	159 (80.7)	151 (77.8)	0.839 (95% CI 0.514–1.37)
Colonoscopy is embarrassing			
Agree	24 (12.2)	29 (14.9)	0.462
Disagree/unsure	173 (87.8)	165 (85.1)	0.789 (95% CI 0.441–1.412)
It is inconvenient for me to see a doctor for CRC screening			
Agree	27 (13.7)	45 (23.2)	0.019
Disagree/unsure	170 (86.3)	149 (76.8)	0.526 (95% CI 0.311–0.89)
Cues to action			
I have attended a public talk on CRC over the last 12 months			
Agree	4 (2)	7 (3.6)	0.377
Disagree/unsure	193 (98)	186 (96.4)	0.551 (95% CI 0.159–1.912)
I have read/heard some materials on TV or newspaper on CRC over the last 12 months			
Agree	125 (63.8)	106 (54.9)	0.08
Disagree/unsure	71 (36.2)	87 (45.1)	1.45 (95% CI 0.96–2.17)
I have heard about CRC from friends or relatives			
Agree	108 (54.8)	96 (49.7)	0.362
Disagree/unsure	89 (45.2)	97 (50.3)	1.23 (95% CI 0.82–1.82)
Doctor recommended CRC screening to me			
Agree	104 (52.8)	71 (36.8)	0.002
Disagree/unsure	93 (47.2)	122 (63.2)	1.92 (95% CI 1.28–2.88)
Friends told me to go for CRC screening			
Agree	46 (23.4)	47 (24.4)	0.905
Disagree/unsure	151 (76.6)	146 (75.6)	0.946 (95% CI 0.594–1.508)
My family told me to go for CRC screening			
Agree	78 (39.6)	67 (34.7)	0.346
Disagree/unsure	119 (60.4)	126 (65.3)	1.23 (95% CI 0.82–1.86)
I would like to be accompanied by my family or friend to see doctor			
Agree	82 (41.6)	58 (30.1)	0.02
Disagree/unsure	115 (58.4)	135 (69.9)	1.66 (95% CI 0.82–2.52)
I am usually accompanied by somebody when I see a doctor			
Agree	73 (37.1)	54 (28)	0.066
Disagree/unsure	124 (62.9)	139 (72)	1.52 (95% CI 0.99–2.32)

**TABLE 4:** Multi‐variable logistic regression model with adjusted odds ratios for predictors for screening amongst doers and non‐doers

	Adjusted OR	95% CI	*p*‐value
Acknowledging that avoiding over‐eating can prevent CRC			
Non‐doer	Ref		0.018
Doer	0.49	0.27–0.88	
CRC is expensive to treat			
Non‐doer	Ref		0.026
Doer	0.47	0.24–0.91	
It is inconvenient for me to see a doctor for CRC screening			
Non‐doer	Ref		0.004
Doer	0.29	0.13–0.67	
Doctor recommended CRC screening to me			
Non‐doer	Ref		0.01
Doer	2.39	1.23–4.63	
I have read/heard some materials on TV or newspaper on CRC over the last 12 months			
Non‐doer	Ref		0.026
Doer	2.16	1.09–4.26	

Most participants were aware of the perceived severity of CRC leading to suffering (91.6%) and death (84.8%). Doers were two times more likely to perceive that CRC will affect their work (OR, 2.26, 95% CI: 1.09–4.93, *p* = 0.04).

Participants were generally in agreement regarding perceived susceptibility of CRC, with the majority agreeing that they were not fated to get CRC (79.7%) and that they can prevent themselves from getting CRC (83.5%). Almost all perceived that CRC screening helps to detect cancer early (96.7%) and early diagnosis will improve survival (97.5%). Similarly, the majority agreed that early treatment can reduce future medical expenses (92.1%).

Within the perceived barriers subscale, respondents were willing to acknowledge the CRC diagnosis (85%), without much fear (71.6%). The odds of non‐doers perceiving that CRC is expensive to treat was twice compared to doers (aOR, 2.15, 95% CI: 1.1–4.2, *p* = 0.026). However, both doers and non‐doers felt that colonoscopy is safe (84.8%), and neither painful (78.7%) nor embarrassing (85.8%). Non‐doers were three times more likely to perceive that it was inconvenient for them to see a doctor to receive a CRC screening evaluation (aOR, 3.44, 95% CI: 1.5–7.89, *p* = 0.004).

Lastly for cues to action, almost all participants denied (96.2%) having attended a public talk on CRC over the past year, but more than half (58.6%) recalled having encountered CRC screening promotional materials on mainstream or print media (TV or newspaper) in the past year. The odds of a doer acknowledging awareness of CRC screening via TV and newspaper channels was two times more likely compared to a non‐doer (aOR, 2.16, 95% CI: 1.09–4.26, *p* = 0.026). Doers were also twice more likely to have received a physician's recommendation to undergo CRC screening than non‐doers (aOR, 2.39, 95% CI: 1.23–4.63, *p* = 0.01). These individuals would also hope to be accompanied by their friends and family when being reviewed by a physician (OR, 1.66, 95% CI: 1.09–2.52, *p* = 0.02).

## 4 DISCUSSION

This is the first local study to analyse the factors predicting compliance towards completing the colonoscopic evaluation in individuals with a positive FIT. Individuals with a positive FIT are already known to possess a higher risk of harbouring colorectal polyps and cancers than individuals who are asymptomatic and tested negative.^17^ Despite possessing high level of CRC awareness and importance of CRC screening, uptake rate for follow‐up colonoscopy remain dismal. Our study therefore identified key factors that could help guide subsequent interventions to improve compliance in these higher risk individuals who are tested positive on FIT and yet not compliant to colonoscopy (i.e. the non‐doers). Structural barriers identified include cost of the procedure and inconvenience which deter positive FIT individuals from going for follow‐up colonoscopy. Decision‐making facilitators such as level of personal motivation augmented by the presence of active promotion by medical physicians, family and friends act as cues to action and encourages follow‐up colonoscopy uptake.

Several broad themes can be identified: The importance of family and close friends for social support, high regard of healthcare professional medical recommendation and the cost and inconvenience of the current processes. In the non‐doer group, it is not the fear of the colonoscopy procedure, nor the worry of knowing the diagnosis, or the assumed embarrassment of the procedure that could account for their non‐compliance. Interestingly, these have always been perceived as a significant barrier.^18^ Effort will need to be placed to explore the extent of financial subsidies required to prompt individuals to seek health promotion behaviour. In addition, the concern over colonoscopy bowel preparation forms a strong deterrence towards initiation and uptake of follow‐up colonoscopy in individuals with FIT‐positive results. There are numerous studies focusing their effort to improve patient satisfaction, tolerance and receptiveness of bowel preparation through the selection of the agent, adjustment of volume required and its frequency.^19^, ^20^, ^21^, ^22^

Instead, when evaluating the results from a socio‐ecological modelling perspective, our findings suggest that the network around these FIT‐positive individuals play a huge role. Family members and close friends are likely to be able to persuade these individuals to complete the colonoscopic evaluation.^23^ The conventional approach through the use of public health campaigns such as public talks may not yield its intended outcome given that almost all the participants reported not attending such public talks. There may be a need to combine the use of mainstream and print media, as well as lay‐person screening advocates (e.g. family and friends), to further improve compliance rates.

It was also clear from our findings that medical professionals are regarded highly, and the advice of trained medical professionals may be integral in converting some of these non‐doers to doers. Currently, the individuals who had a positive FIT would be contacted by a coordinator in each public hospital or sent a letter from the Ministry of Health stating the positive test result, and the recommendation to be seen by a gastrointestinal specialist. Without an answer to the immediate queries that may come to mind––such as the accuracy of the test, assumptions of the aetiology for the positive test and denial of harbouring a cancer––these individuals may belittle or ignore the significant of the positive tests and casually assume it to be due to other minor causations.^18^ Thus, one possible intervention is whether a trained nurse or a doctor should be involved in the dissemination process of FIT results by calling up these individuals with a positive test; hopefully resulting in a higher compliance rate. At the time of writing, the study team is looking into the feasibility of initiating a randomised trial on this research question.

Significantly, although most of the participants were retirees (48.7%), the issue of inconvenience was quite substantial. From the qualitative data obtained, in order to prove that one is healthy, it takes up to 3 days in our local healthcare setting to complete a follow‐up colonoscopy evaluation: (i) pre‐procedure consultation, (ii) the colonoscopic procedure itself and (iii) results dissemination. This is an arduous task and may only pose more challenges to the working population who ironically is the group of individuals who have lesser free time and yet poses as the primary target group (i.e. 50–60 years old) that is more economically active whom we need to recruit more aggressively. However, the study team acknowledges that the questionnaire quantitative findings relating to age and employment status do not possess observed differences influencing their decision to undergo follow‐up colonoscopy. Healthcare providers and administrators will need to look inwards on how to further streamline future processes such as doing same day colonoscopy on the day of consultation and evaluate the role of telemedicine to determine if it leads to time and manpower savings.^24^ Cost is an ever‐challenging topic in a healthcare system like Singapore where co‐payment is still the model of care. Not all patients would be keen to spend money to undergo a procedure which they are sceptical to prove that they are healthy.

The study brings several key strengths. To our knowledge, this is the first large‐scale study in Asia to describe comparisons between compliant and non‐compliant individuals to the CRC screening follow‐up evaluation process after a positive FIT using the HBM. The sample was also derived from a nationally representative cohort, and random sampled to produce two groups (doers and non‐doers) with well‐distributed baseline sociodemographic characteristics. Nonetheless, several limitations exist in our study. This was a cross‐sectional study, and hence the temporal relationship between the effect of psychosocial belief and attitudes on CRC screening could not be elucidated. Given that the responses were self‐reported, there is the possibility that some recall bias may have confounded the findings; participants (especially non‐doers) may also have over‐reported on more socially desirable outcomes. We attempted to mitigate this by training our interviewers to ask questions in an objective and reassuring manner, standardised between both groups. Lastly, despite the robust random sampling strategy, we recognise that our response rate was only 47.8% of the total eligible cohort. We therefore caveat any interpretation of our study's findings with the possibility that individuals who chose not to participate in this study may hold different attitudes, beliefs and perceptions towards follow‐up colonoscopy. Nevertheless, the authors are hopeful that the findings can further educate healthcare administrators and providers on how to further evaluate their current system and workflow, and to consider implementing changes to tackle this neglected yet impactful issue.

## 5 CONCLUSION

Compliance towards completing the screening evaluation process remains hindered by the presence of barriers and misconceptions towards CRC screening. Engagement of family and close friends, and re‐evaluating the internal processes may be helpful to increase compliance towards completing CRC screening in these higher risk individuals. Ultimately, a higher CRC screening compliance rate in any community will enable earlier detection and treatment of CRC and reduce overall healthcare and social burdens for any country.

## CONFLICT OF INTEREST

The authors declare that there is no conflict of interest.

## ETHICS APPROVAL STATEMENT

Ethical approval to conduct this study was provided by the National University of Singapore’s Institutional Review Board (NUS‐IRB; reference code H‐19–075). Verbal informed consent was sought and acquired from all participants before data collection.

## Supporting information

Data S1

## Data Availability

The data that support the findings of this study are available from the corresponding author upon reasonable request.
