# Supplementary material for: Factors predicting improved compliance towards colonoscopy in individuals with positive faecal immunochemical test (FIT)
Source: Cancer Med. 2021 Sep 14;10(21):7735–46. doi: 10.1002/cam4.4275 (PMC8559494; doi:10.1002/cam4.4275)
Supplement: Supplementary file 1 — Data S1 [file CAM4-10-7735-s001.docx]

**Supplementary File 1**

(1) High level of CRC awareness amongst average risk individuals.

*Perceived severity*

"somehow, the, the word death will be… will be very very prominent lah. I think your job will be affected also... And then your income...And generally your health lah. A lot of things you may not be able to do already” (D014)

"Mm… my friend got diagnosed. She had her… half a colon removed, so it affected every area of her life. So she… wasn’t able to continue working. And then she wasn’t able to travel" (D022)

"It will, it will uh, I mean I, stop my earning power and all that, restrict my movement, and uh, furthermore I just gotta be dependent already uh." (D023)

"Stigma, that’s it. The social stigma is there." (D026)

"Then I will worry about my job, ah there's the financial, uh… will I be able to foot the bill and all that lor." (D048)

"I vaguely recall that you know if you have to remove some part of your colon or intestine or something” (ND003)

"Well, the inconvenience because you may have a bag (i.e. stoma) or something, when you have the cancer, you know, depending on what stage it is." (ND042)

*Perceived susceptibility*

"Lifestyle is everything, how you live what." (D013)

"It's 50-50 chances, you know?" (D014)

"But for my case, because I knew they, my dad have it, so I think I better go through lah." (D016)

"Yeah. Maybe you will uh… you don’t eat enough veg, fibre, eating lots of red meat, starch." (D037)

"Uh, I think uh, family history is one. And diet? Especially things like you know, alcoholic beverages, uh, excessively, I mean." (D039)

"The most important is the diet la. So if you, you watch your diet and you take uhhh less meat, and more vegetables and fruits and uhh I think you're safe la! I mean you don't overeat la" (ND002)

"Cos the other would be hereditary right?" (ND012)

"I think… yeah, I think if let's say you… no- never exercise, drink, smoking, drink, then ye- don't eat vegetables, all these would be… yeah, have a big risk ah." (ND023)

*Perceived benefits*

"Early detection, early cure.” (D014)

"Other than to prevent the uh… you have the cancer, then… also at least, I can, I can (inaudible) a peaceful mind that I… my colon is clear." (D020)

"put your mind at ease, yeah.” (D022)

"Uh… benefit is uhm… at least you know that you are not- the part of your body ah, not sick, not health(?)." (D029)

"Well, uh… other benefit is like, you tested, you're okay, then you have uh… peace of mind, and then you don’t think further, then you concentrate to do whatever your things lor." (D031)

"Of course, then you- you have the ease of mind mah (chuckles)." (D033)

"So is of course after screening, then I- they ask you to go. After they clear away the polyps, then safer lah. " (D044)

"You will feel safe- yeah yeah. Then your mood will be happier." (ND023)

"No, the- the thing is the benefit itself, we have a peace of mind lah." (ND032)

*Perceived barriers*

"Yeah, the drink was tough (chuckles). It was three days before that, you already have to curtail your food, you know?" (D014)

"But of course some of them, they don’t like to go through you know, the so-called, surgical risk, of the kind of procedure, the inconveniences." (D016)

"Oh, yes, that is the worst part…Cause of the liquid that we have too drink” (D022)

"then the doctor in this private clinic, he gave something that is like… maybe half the amount less than what is given by the government uh, clinic." (D022)

"Mm… it will… I mean it's a bit troublesome because you have to drink the liquid, you know. All that… it's quite uh… uh… a tedious thing to do lah." (D029)

"Uh, some- well, the thing go in ah, definitely there's bound to be some discomfort, and then your stomach will be bloated. Definitely, there will be discomfort lah. But they also try their best to minimise it lah...you just try to… you- you try to be calm and try overcome(?) it, it will feel bearable lah." (D031)

"Yes, the- the drinking of the water, that- that solution." (D037)

"Uh… that means one whole… for- for one whole day you won't be able to do anything and you just keep drinking water it is actually very uncomfortable." (D040)

"Yeah, because before that, I have to… I have to- cannot eat meat ah, cannot eat what- uh, fi- cannot eat vegetable, is it? Ah, have to procedure(?) also eat uh, lighter food. Then before the actual day, but the medicine that we take ah, is terrible. So I was wonder whether you can invent ah, those things ah. Just- don’t need to take all these ah, just base on blood test ah (laughs)." (D044)

"And then you have to you know it has to be as clean as possible and I think what 6 hours something before the colonoscopy no no no food and drink or something so this inconvenience la” (ND003)

"Just that yeas, visiting a doctor would take, I mean to see the doctor especially government subsidised doctors, you'll have to wait for a long time for" (ND008)

"Ok, barrier 1 would be the need to set aside 2 to 3 days to prepare for colonoscopy, barrier no 2 uhh most people would, ok not most, many people would find the process off-putting- due to the fear of discomfort or embarrassment as well as the need to set aside time" (ND012)

"It's very troublesome la! You already know already what, you have to drink the thing, and then you know it's a you got to wait for the thing to come you know! Like Literally have to sit on the toilet bowl if not you make a mess out of everything!" (ND014)

"Ah…so maybe you put a feedback, this kind of thing (consultation) if they can say no need to pay, just talk, free ah, then I think more people go." (D016)

"Ah… if you're talking about pay below, as I said, pay below $150, some got financial problem, they might not want to go and do all these screening." (D021)

"This is expensive ah? If you have to pay out from your own pocket right, this- it is, there is some insurance and uh… uh… medisave, of course, you won't see the pain ah, you know." (D029)

“Would prefer if it costs less than $500” (D030)

"Uh… my case… I paid around less- I think around 1000, yeah…Actually, it's costly lah…Because even after subsidise also 1k" (D031)

"I have no money. This is expensive…I have no money. This is expensive. Willing to pay less than a hundred for colonscopy” (D032)

"I think it is expensive procedure for those who are not working or the lower income people?" (D038)

"If it's cheap like last time, you can say hor, $5 right, they go for screening, everyone will don’t mind going for screening. But you say $500, how many people can afford?" (D046)

"Lets say some time, every now and then you have to go for the check, then am thinking that it's expensive la, that's for me" (ND011)

*Source of Information*

"Actually, I happened to chance upon this uh, uh, this, what do you call that, uh, the table that they had set up, to collect, to distribute the tool kits. So I just, since it is free" (at the polyclinic) (D014)

"Oh… if you send me the email, some good articles … I don't mind reading them" (D017)

“Newspapers reminded me to go for a test, as well as my wife who helped mr get the FIT KIT” (D019)

"Would it uh… can you advertise it on the TV, all these." and "the media" as "I think nowaday, a lot people using… this kind of Instagram” (D020)

"Easier through TV lah." (D021)

Information to be disseminated "maybe through Whatsapp? Through email? Then media." (D022)

"Yeah, I'm having uh, hard stool, and uh, change in bowel uh, that that was the time, the beginning(?) time uh, change of bowel to, then to visit the GP, GP then asked me to go to polyclinic, and from polyclinic, they referred me to NUH, and that's how the thing started uh." (D023)

"Mm… for me, I'll read newspaper” (D030)

“Prefers roadshows as they are more convincing” (D031)

"Uh, because that time after stool look like (inaudible) red colour, so I also, like… anxious mah. So I, yeah… Besides the bowel changes, so… eh, then I think, it looks like a bit like, the blood, then I just go and take the FIT kit lor. Then all the while, sometimes like like, go to polyclinic, or even some magazine, newspapers, they will show like, all these free kit, where you can get." (D031)

"There's a… a free… I think it's a hea- heavy subsidised… uh, screening offered to me, because of the Singaporeans, yeah.” (D032)

"Oh, written form lor. In pamphlets or whatever, yeah… As long as it's in writing, yeah. Mm, then you can actually uh, slowly read, and then uh… yeah, digest the information and then you take the appropriate action." (D037)

"now almost everyone uh, has handphone what. They can always uh, whatsapp to us. That is easy also, more convenient, you see." (D038)

“Uh… maybe reminder letters from… health organization.” (D040)

"Do in the booklet form, it's like a hardcopy where you can use it as a reference when you think you want to have uh- get some information on it." (D041)

"Oh, maybe just by… newspaper, or when we go hospital checkup that time, you know… just pamphlet lor. Just put there lor, let people see lor." (D044)

"Because at least through- uh, through newsletter, you can at least read it and then uh… you can repeat if you don’t understand, you can repeat it, you see." (D048)

"This and also over TV la! Sometimes people don't read, I would say for me, any form of info.. But then theres people who don't read, then its good to flash on TV to remind people about colo colon cancer la cos Cos some people don't read, they watch TV so that kind of news also can infiltrate, I mean uh the person la” (ND001)

"Maybe… Like ok you know like SMS reminders uhhh, I don't really remember whether if is there such a thing now, if not I think SMS reminders to say you know what uhh go collect your FIT kit for 2020 that kind of thing la (chuckles)" (ND003)

"Ohh, I think I think for me is I.. I.. uhh would like to watch TV programs so like from the TV programs would be better for me" (ND008)

"Mmmm, maybe just throught the advertisement newspaper or advertisement la” (ND011)

"Like so yea because nowadays it's so convenient to get information online. Uhhh, I supposed emails? If there is a way to send information about colorectal screening? Through emails, uhhh perhaps whatsapp" (ND012)

"Right? Ya, so ya. And the the other forms of mass media, reading TV radio or papers, I supposed they will reach out to other people as well, so I believe uhh yea all forms of mass communication uh will be helpful yea cos every person will be reached differently right" (ND012)

"Newsletter of course is quite good… more expensive and more manpower. But TV of course very expensive." (ND023)

“I read magazine, I read news online." (ND027)

"So if they were to do a roadshow every six months, or every three months, targeting at the certain age, or a certain group of people, then that would be fine!" (ND032)

**(2) Health seeking behaviour determined by degree of personal motivation.**

"It is uh… it is a matter of priority mah, what's important to you mah." (D013)

"I mean since there discovered it, I think it's better for me to go." (D014)

"But personally is that, for me is that, there is a, you know, free screening, you know, it's better for me to know ah. To know early ah, whether you got it or you don’t get it, or what stage you are having it ah. So I think to me, personally, for my case ah." (D016)

"Uh… make me decide, I sent my blood sample, I mean my stool sample to… they found that… something is wrong, so I must go for the checkup." (D021)

"Ah... As I want to know my health." (D046)

"Uh, if I find that something is not correct with the faeces or what ah, then I will go lor." (D048)

"Mm you see, yeah yeah, why should I… it is not necessary, you see." (D021)

"No, if it is, if it concerns my life and you know, if they charge, I pay!” (D023)

"But for me, same thing. Even one thousand, I woul still pay. It's my health, it's- yeah. Great(?) life, I always have to be responsible." (D031)

"No matter the costs the fees, for the peace of mind that you must have" (ND001)

"Uh… nope. I thought I said uh… unless I feel something wrong, I will go for a check up." (ND027)

**(3) Importance of social support and medical professional advocacy.**

"Yeah. Yeah so your emotional wellbeing lah, when you hear that you're being the family will be affected, because the family will need to be, sort of the main support pillar," (D014)

"Through the GP uh, For example, in my case, it is the GP knows that there is something, tell me, hey go and get the free, you know." (D016)

"Mm… it would probably like uh, when I feel uncomfortable uhm, or… I suspect myself having some sort of illness,- and with the encouragement from uh, family members, like I will go." (D040)

"When the doctor uh… tells me the seriousness of it." (D046)

"So you hear of friends having colon and the public talk and all that, as in of course it will prompt you to go for my colonoscopy la." (ND001)

"Like Ive said, if I don't feel that I'm uncomfortable, I think that (inaudible) I don't intend to go unless somebody recommended, and I know my friend that got colorectal he died within one and a half years, same with my father one and a half years but I dont know how come I thinking that I'm healthy, I don't feel like going” (ND011)

“Well, I supposed it's a a you know when the screening gives the person peace of mind and it also assures family members" (ND012)

"I think newspaper, yeah will help popularise the information. But the accurate part, I'm not quite sure you know, the reporters will report it accurately, and that’s- that’s what we would consider, got the professional side and the- you know, the reporters, yeah they have different skills." (ND028)

"I think reach out, the only way… you go to where the regular polyclinics checkup, from there…then they will advise you, you have to go ah." (ND032)
